# “It’s just us sitting there for 23 hours like we done something wrong”: Isolation, incarceration, and the COVID-19 pandemic

**DOI:** 10.1371/journal.pone.0297518

**Published:** 2024-02-14

**Authors:** Alana Rosenberg, Lisa B. Puglisi, Kathryn A. Thomas, Alexandra A. Halberstam, Rosemarie A. Martin, Lauren Brinkley-Rubinstein, Emily A. Wang

**Affiliations:** 1 SEICHE Center for Health and Justice, Department of Internal Medicine, Yale School of Medicine, New Haven, Connecticut, United States of America; 2 Department of Behavioral and Social Sciences and Center for Alcohol and Addiction Studies, School of Public Health, Brown University, Providence, Rhode Island, United States of America; 3 Department of Population Health Sciences and the Samuel Dubois Cook Center on Social Equity, Duke University, Durham, North Carolina, United States of America; Kore University of Enna: Universita degli Studi di Enna ’Kore’, ITALY

## Abstract

For the millions of people incarcerated in United States’ prisons and jails during the COVID-19 pandemic, isolation took many forms, including medical isolation for those sick with COVID-19, quarantine for those potentially exposed, and prolonged facility-wide lockdowns. Incarcerated people’s lived experience of isolation during the pandemic has largely gone undocumented. Through interviews with 48 incarcerated people and 27 staff at two jails and one prison in geographically diverse locations in the United States, we document the implementation of COVID-19 isolation policies from the perspective of those that live and work in carceral settings. Incarcerated people were isolated from social contact, educational programs, employment, and recreation, and lacked clear communication about COVID-19-related protocols. Being isolated, no matter the reason, felt like punishment and was compared to solitary confinement—with resultant long-term, negative impacts on health. Participants detailed isolation policies as disruptive, detrimental to mental health, and dehumanizing for incarcerated people. Findings point to several recommendations for isolation policy in carceral settings. These include integrating healthcare delivery into isolation protocols, preserving social relationships during isolation, promoting bidirectional communication about protocols and their effect between facility leadership and incarcerated people. Most importantly, there is an urgent need to re-evaluate the current approach to the use of isolation in carceral settings and to establish external oversight procedures for its use during pandemics.

## Introduction

COVID-19 had a devastating effect on carceral facilities in the United States (U.S.). They were sites of early cluster outbreaks [1], and at the peak of the pandemic, incarcerated people were 5 times as likely to be infected with COVID-19 and 2.7 times more likely to die from COVID-19 than the general population [2]. Despite this heightened risk, carceral settings were not fully integrated into pandemic public health responses (*e*.*g*. robust testing and vaccination) [3] and as a result, many carceral settings experienced perpetual “outbreaks,” largely managed with policies that lead to isolation [4].

Pandemic-related isolation in carceral settings has primarily taken three forms: 1) medical isolation for those sick with COVID-19, 2) quarantine for those exposed or whose infection status is unknown, and 3) en masse isolation through facility-wide lockdowns. The Centers for Disease Control and Prevention (CDC) defines medical isolation as “the physical separation of an individual with confirmed or suspected COVID-19 infection to prevent their contact with others and reduce the risk of transmission.” In carceral settings, medical isolation includes both single-cell and cohorted isolation, where people with confirmed COVID-19 infection are housed together. Quarantine, defined by the CDC as “the practice of separating individuals who have had close contact with someone with COVID-19 to determine whether they develop symptoms or test positive for the disease” [5] was implemented following admission to observe incoming individuals for symptoms of illness and keep them apart from other incarcerated people. Finally, unique to carceral settings are facility-wide lockdowns, in which incarcerated people are held in their cells or dorms for more than 22 hours a day and where access to common areas, recreational activities, and social contact was eliminated. Carceral facilities normally implement lockdown in response to security threats but have employed this practice as a means of both minimizing viral transmission and responding to COVID-19-related staff shortages.

Despite the ubiquity of these practices during the pandemic, data on the number of people isolated and the duration of their isolation are largely unavailable. According to the COVID-19 Prison Project, only nine state prison systems (facilities which typically house people who have been convicted and are serving sentences of more than a year) routinely provide quarantine data [6], seven provide data on medical isolation [7], and only five publicly post lockdown policies [8]. Even less is known about jails (facilities which house people who are unsentenced or serving sentences of less than a year). Media reports, however, indicate that dangerously lengthy isolations have been a pervasive experience of incarceration during COVID-19. For example, one prison in Colorado documented successive quarantine periods that averaged 50 days each and provided incarcerated people with just 20 minutes out of their cell time, four times a week [9]. The Washington DC jail, which houses 1500 people, the majority of whom are unsentenced and awaiting court dates, locked down the facility for almost 400 days, with only one hour a day of out-of-cell time [10].

These forms of isolation approximate conditions of solitary confinement, commonly used to punish incarcerated people by leaving them in their cells for over 22 hours a day. It is a practice known to have significant and lasting negative impacts on mental and physical health. Solitary confinement exacerbates existing mental illness [11] and can lead to new psychological problems, including post-traumatic stress disorder [12], anxiety, depression [13], psychosis and paranoia [14]. Solitary confinement also increases risk of self-harm and suicide [15] and physical health issues, including possibly hypertension, heart attacks [16], and premature death [17]. These health consequences are long-lasting and persist even after people are removed from confinement [18]. Because of these documented consequences, the United Nations Standard Minimum Rules for the Treatment of Prisoners recommends limited use of solitary confinement for as short a period as possible and prohibits prolonged use, defined as greater than 15 days [19], though these standards have not been adopted in most U.S. carceral systems.

Despite the health harms of extended periods of isolated confinement, very few empirical studies document incarcerated people’s lived experience of isolation during the pandemic. This gap is due, in part, to visitation bans enacted during the COVID-19 pandemic, which exacerbated difficulties of conducting research in prisons and jails. A few qualitative studies in European prisons during the pandemic described decreased communication, detachment from ordinary life within and outside of prison, and increased distance from family [20]; a deepening of depression, anxiety, or risk of self-harm [21]; and negative emotional associations as a consequence of isolation within carceral facilities [22].

Even fewer studies of isolation policy and health effects in U.S. carceral settings exist. Liu and colleagues surveyed 788 incarcerated people at four northern California jails, finding 38% of incarcerated people had worse mental health because of decreased family contact and changes in recreation and programming [23] To our knowledge, the only qualitative study of isolation during the pandemic in the U.S. documented the views of 31 men already in highly restrictive housing, but focused on perceptions of COVID-19 risk rather than the experience of isolation [24]. In a scoping review of published articles, Johnson and colleagues document the consequences of isolation policies in carceral systems but note a lack of empirical studies on their impact on health [25]. As far as we know, no study has described incarcerated people’s and correctional staff’s perspectives of COVID-19 isolation policies and their impact on mental health and wellbeing.

This study responds to the dearth of data on the effect of isolation during the COVID-19 pandemic on incarcerated people and correctional staff health [26]. We describe how incarcerated people at three U.S. carceral facilities experienced various forms of isolation and how it affected their health, as well as how correctional staff perceived conditions of isolation.

## Methods

### Setting

This qualitative study was conducted as part of a larger study focused on COVID-19 prevention and treatment in carceral systems. We interviewed incarcerated people and correctional staff at a rural jail, an urban jail, and a state prison located in an urban area. The facilities were located in three different states in the U.S., each with significant COVID-19 outbreaks early in the pandemic. The facilities chosen were a convenience sample of carceral institutions identified through our existing networks that were open to collaboration, and specifically, providing data on testing and other mitigation strategies to manage COVID-19. We sought and obtained participation from facilities located in both rural and urban settings, and representation of both jail and prison settings. Interviews took place between April 2021 and April 2022. Correctional leaders and medical staff publicized the study through email, word of mouth, and flyers posted in the facilities. Incarcerated individuals let correctional or medical staff know of their interest; correctional staff expressed interest to a point person at the facility. Screening and the consent process took place immediately before the interview. Participants were eligible for the study if they spoke English, were 18 years or older, and could demonstrate an understanding of the study (see description below).

Incarcerated participants were screened and interviewed in a private room without the presence of correctional or medical staff. Interviewers informed participants of benefits and risks of participating, and participants gave verbal consent. Potential participants’ capacity to consent to the research was assessed using the Teach-to-Goal protocol [27] This protocol involves asking potential participants to describe the research goals and procedures after they have been explained during the consent process. Interviewers were trained in the consent process and Teach-to-Goal protocol. Participants were told they could opt out of the study at any time. The participation of incarcerated participants did not affect their treatment while incarcerated, nor did correctional staff’s participation affect their terms of employment. The project was approved by Yale University’s Institutional Review Board, the local facility or county government, and the federal Office of Human Research Protections.

Incarcerated people who participated were compensated $50 through their carceral facility account, a gift card sent to a family member or friend, or a gift card given at time of release, depending on the recipient’s choice and facility rules. Staff also received $50 in the form of an electronic gift card. In the absence of universal guidelines on compensation for incarcerated research participants [28, 29], we chose to compensate $50 per interview for all participants to regard and protect the dignity and rights of incarcerated people equally to those of staff. This strategy was approved by the Yale Institutional Review Board, which deemed the financial incentive not of a magnitude that would impair the incarcerated person’s ability to weigh the risks of the research against the value of the financial incentive [30]. The financial incentive was also approved by each facility.

These findings about the experience of isolation and implications for health and wellbeing emerged as we analyzed data on COVID-19 mitigation strategies. A separate analysis of this dataset on the topic of COVID-19 mitigation strategies has been published previously [31]. The current analysis is based on interviews with 48 incarcerated people, 17 correctional officers, and 10 correctional leaders. At Site 1, we interviewed 16 incarcerated people, 9 correctional officers, and 5 correctional leaders. At Site 2, we interviewed 17 incarcerated people. Four participants at Site 2 were interviewed a second time to probe specific themes. Correctional leaders and officers did not participate at Site 2 due to lack of union approval. At Site 3, we interviewed 15 incarcerated people, 8 correctional officers, and 5 correctional leaders. In total, 79 interviews with 75 participants were included in this analysis. See Table 1 for demographic information for participants.

**Table 1:** Demographic information for participants.

	Incarcerated People (48)	Personnel (27)
Average Age	41	43
Gender		
Male	35	22
Female	13	5
Race		
African American	15	2
Asian	1	2
Native American	3	1
Native Hawaiian or Pacific Islander	1	0
White	21	20
Other	7	1
Prefer Not to Answer	0	1
Ethnicity		
Hispanic/Latinx	9	1
Not Hispanic or Latinx	38	26
Prefer not to answer	1	0
# years living or working at facility		
0–5 years	38	10
6–10 years	2	3
11–15 years	1	1
More than 15 years	7	13

### Data collection

Given COVID-19 restrictions, interviews were conducted via videoconferencing. Interviews were semi-structured and averaged 50 minutes in length. Each interview was conducted by one of three interviewers who represented the disciplines of public health, internal medicine, and psychology and included a formerly incarcerated person and a person affected by the incarceration of a family member. The interview guide covered: history at the facility, job responsibilities, general COVID-19 experience, medical care including COVID-19, and experience with COVID-19 testing and vaccines.

Participants shared experiences of isolation in response to the question, “How are things different now than before? Are there any changes? (probe for changes to visitation, amount of isolation/lockdown)?”, as well as questions about COVID-19 testing, vaccination, treatment and other COVID-19 mitigation strategies at the facility. For facility leaders, COVID-19 decision making processes were also probed (see S1 File for full interview guides). Interviewers sought to understand participants’ COVID-19 related experiences, and the meaning they assigned to such experiences through probing and following cues to determine topics of priority for participants. Interviews were audio-recorded. We transcribed each audio file and uploaded the transcript into Dedoose, a qualitative data management software platform.

### Data analysis

We sought to understand the lived experience of isolation in carceral settings and the meaning that this experience held for participants. This approach guided our thematic analysis [32]. A multidisciplinary team including the interviewers, other team members, and students applied a tiered coding approach, first developing and applying index codes and then thematic codes [33]. Index codes related to isolation included individual isolation, unit isolation, and movement to and from isolation. Once we applied the index codes to all transcripts, we wrote detailed analytic memos and, simultaneously, further sorted the indexed material into newly developed thematic codes (e.g., isolation as punishment). Each transcript was coded by at least one student and one staff team member. Differences in the application of codes were discussed until consensus was reached. Sometimes codes were redefined for clarification, and revised codes were then applied to previously coded transcripts. We examined themes across the different participant roles interviewed for the study (e.g., incarcerated person, correctional officer), as well as the different facilities.

## Results

Participants understood the need for protection from the virus through isolation. Yet the context within which isolation occurred—including limited recreational opportunities, unclear communication about COVID-19 protocols, and extremely limited contact with family members and friends—was detrimental. Incarcerated people experienced all forms of isolation used during COVID-19 (medical isolation, quarantine, and lockdown) as punishment, deeply disruptive, detrimental to mental health, and dehumanizing. Correctional staff accounts endorsed that incarcerated people experienced isolation as punitive, disruptive, and detrimental to mental health.

### Conditions of isolation

Before elucidating the themes of our analysis related to the experience of isolation, we detail the conditions of isolation, as described by participants, to give context to the themes described later. Despite the varying geographic regions and the combination of jail and prison facilities, participants expressed similar conditions of isolation. Participants reported loss of usual routines that allowed for out-of-cell time, extreme informational and social isolation, and facility-wide policies that fluctuated with COVID-19 waves. These conditions characterized and deepened experiences of isolation.

#### Lack of recreation

To prevent COVID-19 infections, educational, religious, and recreational programming were largely halted at the onset of the pandemic. Exercise facilities, libraries, barber shops and yards were inaccessible. Facilities served food in cells rather than in dining areas. All employment for incarcerated people—except for essential positions—was eliminated. Programming, facilities, and employment opportunities were re-opened during periods of low levels of COVID-19 transmission, only to close again during periods of high risk of COVID-19 and accompanying staff shortages. One incarcerated person explained:

“It was really hard adjusting, going from having… recreation all the time to being locked down most of the time… being able to work out whenever you wanted to working out a couple times a week if you got the chance… There was a point where we couldn’t even go outside.” (IP20)

One incarcerated person described constant changes in recreation access as a frustrating and unpredictable “little dance” that lasted over a year. As COVID-19 cases dropped, things slowly reopened, only to return to full lockdown:

“That was so frustrating because for a second, we kind of felt like we were getting a little bit more. I’m talking about recreation…fresh air, simple shit. Just walking and stretching your legs…you’re out there walking around and then you feel like, boom, okay. We’re getting back to normality and then, ‘All men return to your cell. Emergency count.’”(IP0030)

Correctional officers mentioned several specific hardships facing incarcerated people in isolation, including a lack of exercise, community, commissary, and the ability to eat in the cafeteria. Thus, correctional officers understood the immense challenge isolation posed for incarcerated people. Correctional leaders struggled to provide adequate recreation and gym time to incarcerated people due to the need to keep units quarantined to avoid cross contamination, as well as staffing problems from COVID-19 absence. A correctional leader said, “As a product of staffing levels and limiting the interaction between inmates, we’ve changed the way that inmates are scheduled in the jail, so that they’re locked down more hours of the day” (CL007).

#### Lack of clear COVID-19 communication

Isolation occurred against a backdrop of confusion about its reasons, length, and degree of risk that COVID-19 infection posed. Incarcerated participants reported minimal communication about community and facility COVID-19 rates. At one site, participants learned about an outbreak at their facility through the local news rather than from facility authorities. One incarcerated person stated,

“We were watching the news, reading the newspaper. We were getting little tidbits here and there out of it, but still nothing had come out that there was COVID-19 in the [facility] yet. And then all of a sudden when it did, there was 130 cases I believe that hit all of a sudden… We caught it first on the news before we heard it from staff.” (IP010)

While this incident happened early in the pandemic, communication remained a challenge throughout. As the pandemic continued, constantly changing protocols and insufficient messaging, such as reliance on ineffective email communication between staff members, left both staff and incarcerated people lacking clear understanding of risk and mitigation strategies. One staff member reported, “Honestly, [the policy] changes so often I don’t even know what’s going on. I feel like I get two or three emails every day about changes.” (CO013)

#### Reduced communication with family and friends

Long halts to in-person visitation and limited opportunities for phone calls and video visits with family and friends amplified isolation. Facilities initially offered free phone calls and video visits to mitigate isolation, but later reinstated fees. During lockdowns, phone calls, which were normally available multiple times a day, became available only once a day during the brief out-of-cell period. Incarcerated participants reported having to choose between a shower or phone call due to limited out-of-cell time. An incarcerated person stated, "[I]t was a choice. Do I call a loved one and say, ’Hey, I’m okay,’ or do I go and, you know, and wash up, practice my hygiene to remain healthy?" (IP023). Incarcerated people also expressed frustration that family members could not get health information. One incarcerated person said: “When I was locked in quarantine my mom called up here, and they wouldn’t give her any information about whether I was positive or not” (IP020). Some participants suggested that they would like family members to receive medical updates.

### Experiences of isolation during the COVID-19 pandemic

While a few participants expressed a recognition of the importance of certain forms of isolation for protection from COVID-19, most participants experienced the implementation of isolation, whether medical isolation, quarantine or lockdown, as punishment rather than care or protection; deeply disruptive to social connectedness; detrimental to mental health; and dehumanizing.

#### Need for isolation

Some participants described isolation as necessary in certain circumstances for protection from COVID-19. As one participant said, isolation was put in place “so there wouldn’t be any further infections” (IP023). Describing quarantine procedures for those first entering the facility, one incarcerated person said of the facility: “They do the extra processes on keeping the people more safe” (IP012). These statements primarily referenced the general purpose of isolation. One participant, however, expressed a direct link between the isolation of another incarcerated person and their own protection from illness: “good thing we wasn’t around him” (IP042).

#### Isolation as punishment

While isolation was ostensibly meant to protect, isolation often resembled solitary confinement and was seen as a form of punishment. Incarcerated people explained medical isolation with the phrase “the hole,” a term for the cell used for prolonged solitary confinement: “It’s basically as if they’re in a hole for a violent action or something” (IP015). Another incarcerated person described a friend asking for a cough drop and being sent to the “hole” (IP033). Yet another conveyed the injustice of punishment-like conditions for illness:

[P]eople that test positive shouldn’t feel like they’re being punished when they’re just sick. They shouldn’t be locked away in a room by themselves with no TV, no way to contact the outside other than mail…They should be able to watch TV… shower

… have a coffee if they want it … make themselves a soup…” (IP020).

Incarcerated participants described experiencing lockdown as punishment as well. One incarcerated person described lockdown: “It’s just us sitting there for 23 hours like we done something wrong” (IP037). Correctional staff also described the connection between isolation and a sense of punishment. One staff member stated:

“[I]f we have to lock the facility down… everyone in the jail was getting out for one hour… you normally give your worst inmates, the people who are most violent, the people who are most problematic, 23 hours locked into a cell, one hour out of the day…and we’re doing that to everyone” (CO011).

#### Isolation as deeply disruptive

For many incarcerated participants, being relocated to medical isolation felt like losing one’s own space and accompanying familiarity with their cell, roommates, neighbors, and unit. Such situations are unique to carceral settings where ultra-local social dynamics significantly influence sense of safety and predictability of daily life [34]. Participants expressed anxiety anticipating a move, the disruption of the move itself, and the need to readjust to a new space during and after isolation or quarantine. An incarcerated person explained:

“I was worried…that I had to leave the comfort of—it’s hard for you to imagine because it’s a cell to you…but when you’re comfortable in a certain spot and…pulled out of it and you’re going to another place where you might be bunked with a guy that you don’t even know…it just seemed like a very stressful situation to be in….that was a very anxious feeling” (IP030).

Such moves disrupted relationships incarcerated people had spent years building. In some cases, after isolation, individuals did not return to the same cell or unit. Adjusting to a new place sometimes heightened the risk of disciplinary infractions because of the need to acclimate to new corrections officers and incarcerated people. As one incarcerated person described:

“So what you were used to for years, you have to now change it, because there’s a new officer…new inmate, in an enclosed environment like a certain block. That can change your whole sentence…there’s new components you add to your life… Any given day they could be having a bad day and you don’t know them… You just have to get used to everybody and know people’s tendencies and mannerisms and you move accordingly” (IP032).

In some cases, resistance to testing ensued because of the disruption that isolation caused. One incarcerated person explained that they and others in their unit, after witnessing positive tests lead to removal to quarantine, refused to test en masse. This led to quarantine of the entire unit, together.

"We all got together and we all talked to each other and we said, ‘Hey, next time a CO comes in here and asks us for this COVID-19 [test],’ cause they would come every week, ‘we’re gonna tell them we’re not taking the test.’…we don’t see the point of taking that test, being moved out of a comfortable situation now into a hostile situation” (IP015).

Correctional officers, too, noticed that community was disrupted with isolation in place. One correctional officer said:

“They’re not getting the community, which may seem weird to say in jails or whatnot, but they do build communities in inmates [and]….the inmates get to know deputies or correctional officers pretty well. So there is that community and [isolation] limits a lot of that, where it can really weigh on people’s mental health” (CO02).

#### Isolation as detrimental to mental health

Participants described isolation in all forms as detrimental to their mental health, exacerbating existing mental illness and creating new stressors. As lockdown conditions improved in one facility, an incarcerated person stated: “They started recently just letting up a little bit–the restrictions, but it was bad. It was bad. I was really stressed out, really depressed too….…It’s just so many feelings going on in your head at the same time: anger, aggravation, stress, depression, all that. All that in one” (IP024). These feelings were worsened by restrictions on recreation, which the same participant described as a way to “keep your mind occupied.” Lack of communication with family and friends also exacerbated stress.

Some correctional officers also noted the impact of isolation on the mental health of those who were incarcerated. A correctional officer reported that lockdown resembled solitary confinement and “[t]hat’s not good for anyone’s mental health, especially the fact where our mental illness population has climbed during this time. So if you have someone who’s already mentally ill, and then they’re locked in 23 hours a day, they deteriorate rather quickly…. it has not been healthy for them” (CO011).

Access to mental healthcare was limited during COVID-19. At one facility, unless someone was experiencing a crisis, regular mental health appointments were postponed or shortened during medical isolation and quarantine. One incarcerated person said:

“I’ve been told I’ve had an appointment for the last two months and I haven’t seen anybody… I’ve always had depression and anxiety and bipolar disorder, but my anxiety has been skyrocketing… and I’ve asked for help and I haven’t received any” (IP034).

Most participants did not feel that their mental health needs were adequately addressed during the pandemic, despite increased need.

#### Isolation as dehumanizing

Many incarcerated participants reported feeling dehumanized by isolation, especially as it exacerbated their feeling of powerlessness. Some reported the devastation of being treated as “contaminated” while sick. One incarcerated person suggested that the actions and attitudes of correctional officers contributed to this feeling: “They should treat them like they have an illness, and not like they’re a caged… animal” (IP024). Limited access to showers and food delivered on Styrofoam trays through a flap in the door were also experienced as dehumanizing. An incarcerated person stated, “I felt like I was… infested with something so disgusting that nobody wanted to touch me” (IP018).

A similar sense of dehumanization resulted from perceived insufficient treatment during COVID-19 illness. One incarcerated person said, “I didn’t feel like they was doing what they should have been doing to make sure like people don’t die. It’s like they just [said]…’I hope you survive’” (IP033). Another explained, “Medical care while I was in the isolation unit was pretty scarce” and that they saw the nurse only three times during their 12 days in quarantine (IP034). A third stated, “You just go through it…you’re either gonna live or you’re gonna die” (IP007). Others expressed a desire for correctional and medical staff to provide a feeling of being cared for even when specialized treatments were unavailable. Incarcerated participants also expressed the need for, but lack of, care for post-COVID-19 symptoms.

Incarcerated participants talked of the lack of showers and clean clothing as dehumanizing. One said, “They gave me a little container I could wash up with, but… couldn’t shower… That was the worst part of it really is the not showering” (IP016).

## Limitations

Our study has several limitations. Facilities were a convenience sample of institutions known from our researcher network that were open to collaboration. COVID-19 policies and the way they were implemented at these institutions, generally and with respect to isolation, may not include the full array of responses within carceral facilities. Therefore, the experiences expressed here may differ from experiences at other institutions. We were unable to interview correctional staff at Site 2 due to lack of approval from union leadership. The supplemental perspective from correctional staff from Site 2 might have shed light on dynamics particular to that facility. Interviews were conducted virtually. In-person interviews may have revealed physical reactions to questions that would have led to different follow up questions. Finally, while efforts were made to give incarcerated people privacy during the interview through private interview rooms, participants may have still felt surveilled during interviews given the nature of carceral facility rules, which allow correctional staff to record, listen to, and read the private communication of incarcerated people.

## Discussion

The pandemic created new forms of prolonged isolation in carceral settings that reduced or eliminated recreational opportunities, diminished opportunity to communicate with family and friends, and created a culture of misinformation regarding COVID-19. Incarcerated people experienced medical isolation and quarantine, which were intended as a form of public health protection, as punishment, akin to solitary confinement. Isolation led to disruption of a sense of community, safety and security. Incarcerated individuals expressed deleterious mental health effects and felt dehumanized.

These findings point to several recommendations for safeguarding the wellbeing of those that live and work in carceral facilities during respiratory infectious disease outbreaks: ensuring adequate physical and mental healthcare delivery to incarcerated people in isolation, preserving relationships throughout isolation, improving communication between facility leadership and incarcerated people, and limiting and regulating the use of isolation. These recommendations regarding isolation, described in the following sections, complement broader ones stemming from these interviews and published separately [31].

### Integration of healthcare delivery into isolation conditions

If medical isolation is to promote health, healthcare delivery should be more fully integrated into isolation settings. Those in medical isolation must be visited regularly by medical staff. Treatments available to incarcerated people should be on par with treatments available in the community, and mental health care should be more, not less, accessible during isolation, given the risk of aggravating mental illness or increased mental health symptoms during periods of isolation. Suicide is the leading cause of death in jails and fourth leading cause in prisons in the United States [35], and isolation is a known risk factor for suicide in carceral settings [15, 36]. Mental healthcare capacity of facilities should be scaled up to meet need, and virtual appointments should be utilized for care during periods of isolation. Further, our findings support existing recommendations that each facility’s population should be limited to its medical care capacity [37].

### Preserving relationships through isolation

Isolation policies in carceral settings should take into account the centrality of relationships to health. Isolation should occur in cohorts as much as possible [5]. Incarcerated people placed in isolation should be able to freely communicate with family and friends through telephone and virtual visits; the costs of telephone calls and virtual visits should be eliminated, and facilities should procure equipment to enable such communication (e.g. tablets). Further, family notification protocols (with prior consent from the individual) should be implemented in case of illness. As much as possible, individuals should be allowed to return to previously assigned housing after isolation, to ensure continuity of social support via established relationships between incarcerated people.

### Communication between facility leadership and incarcerated people

Findings point to the importance of bidirectional communication between facility leadership and incarcerated people to avoid informational isolation or misinformation. Facility leadership should communicate isolation-related protocols, reasons for their implementation, and changes in protocols over time. Communication should include level of infection risk, current variants, virus transmission pathways, and duration of isolation. Likewise, there should be a mechanism for incarcerated people to provide input on the lived experience of isolation to inform facility decision-making.

### Limiting and regulating isolation

Finally, our study suggests that, given the similarities between ways in which all forms of isolation during COVID-19 resembled solitary confinement, regulations defining isolation conditions and limiting its use must be implemented and enforced immediately. There is an abundance of evidence that solitary confinement aggravates multiple health issues including persistent and long-lasting mental illness [11–14], physical health issues [16, 17] and as noted earlier, increased suicide risk [15, 36]. In reviewing COVID-19 mitigation strategies, researchers have raised concerns about the degree of isolation and the risk of violating human rights of incarcerated people during the COVID-19 pandemic [38]. Findings from the current study support the idea that limiting isolation is important for preserving mental health. It also may improve COVID-19 testing as a mitigation strategy, as people sometimes resisted testing to avoid medical isolation. The following strategies for isolation regulation during pandemic times would go far to ensure that disease prevention is maximized, and health harms minimized.

First the United Nations’ Standard Minimum Rules for the Treatment of Prisoners, also known as the Mandela Rules, should be upheld, even in times of pandemic. These rules prohibit isolation of 22 hours a day for more than 15 days [19]. Prolonged lockdowns of more than 15 days is not appropriate even during times of pandemic. Furthermore, incarcerated people enduring all forms of isolation—medical, quarantine, and lockdown—should have access to resources and activities available on regular housing units, including television, tablets, radio, reading materials, clean clothing, and showers [34]. In fact, we know that very little COVID-19 was spread outdoors [37] and increasing outdoor time may be a safe strategy to improve mental health.

Second, carceral facilities should be mandated to release details on conditions of all forms of isolation, including medical isolation, quarantine, and lockdown, the number of incarcerated people subjected to them, and duration of each incident of isolation. Without strict reporting requirements, the degree of isolation implemented in carceral settings is unknown. For example, the Arthur Liman Center for Public Interest Law and Correctional Leaders Association recently reported that the number of people held in restrictive housing and the length of its duration has decreased since 2015, based on a voluntary self-reported survey of facility leadership. The report raises concerns that survey responses do not capture COVID-19 lockdowns and isolations [39]. Relatedly, research partnerships that allow investigation into all forms of isolation and its immediate and long-term effects are important to understand conditions to which more than one and a half million people in the U.S. are subjected. Research at state prisons and local jails, government-funded institutions, particularly about the lived experience of isolation, should be considered necessary and conducted regularly.

Third, external oversight bodies must have power to enforce these regulations, as detailed in the Mandela Rules. While the U.S. Government Accountability Office provides oversight of federal prisons, oversight of state jails and prisons is inconsistent and varies widely by state and jurisdiction. Currently, no system offers sufficient oversight. As is practiced in other countries [40], implementing a consistent oversight board or Ombudsman’s office to oversee jails and prisons in the U.S. is a crucial step to ensuring humane treatment of incarcerated individuals, especially during pandemics.

COVID-19 has made isolation experience in carceral settings ubiquitous, and its health impacts are likely long lasting and broad reaching. More longitudinal research is needed to understand these impacts and inform carceral pandemic policy. When indicated, medical isolation and quarantine should be overseen by medical personnel, implemented cautiously, and with several protections to safeguard incarcerated persons’ health and humanity, including continued contact with friends and family, access to mental and physical healthcare, recreational and educational supports, and information about the length and reason for isolation. Prolonged facility lockdowns as a response to COVID-19 related challenges are deeply harmful to the health and wellbeing of incarcerated individuals. Such lockdowns must cease in order for U.S. prisons and jails to comply with internationally recognized standards.

## Supporting information

S1 File Interview guides. (DOCX)

## References

[1] Terebuh PD, Egwiekhor AJ, Gullett HL, Fakolade AO, Miracle JE, Ganesh PT, et al. Characterization of community‐wide transmission of SARS‐CoV‐2 in congregate living settings and local public health‐coordinated response during the initial phase of the COVID‐19 pandemic. Influenza and other respiratory viruses. 2021 Jul;15(4):439–45. doi: 10.1111/irv.12819 33058538 PMC7675529

[2] Marquez N, Ward JA, Parish K, Saloner B, Dolovich S. COVID-19 incidence and mortality in federal and state prisons compared with the US population, April 5, 2020, to April 3, 2021. JAMA. 2021 Nov 9;326(18):1865–7. doi: 10.1001/jama.2021.17575 34613335 PMC8495600

[3] Klein M, Kowalski MA, Woo Y, Solis C, Mendoza M, Stohr MK, et al. The novel coronavirus and enforcement of the new separate system in prisons. Criminal Justice Policy Review. 2022 Mar;33(2):206–30.

[4] Hagan L. Updates to CDC COVID-19 Guidance for Correctional and Detention Facilities [Internet]. 2022 [cited 2022 Oct 13]. Available from: https://www.cdc.gov/coronavirus/2019-ncov/science/community-levels.html

[5] Centers for Disease Control and Prevention. Guidance on Prevention and Management of Coronavirus Disease 2019 (COVID-19) in Correctional and Detention Facilities [Internet]. 2022 [cited 2022 Oct 13]. Available from: https://www.cdc.gov/coronavirus/2019-ncov/community/correction-detention/guidance-correctional-detention.html

[6] Maner M, LeMasters K, Lao J, Cowell M, Nowotny K, Cloud D, et al. COVID-19 in corrections: Quarantine of incarcerated people. PloS one. 2021 Oct 5;16(10):e0257842. doi: 10.1371/journal.pone.0257842 34610015 PMC8491943

[7] Maner M, Behne MF. Medical isolation in prisons: Definitions and dissonance. In: Academic & Health Policy Conference on Criminal Justice, Academic Consortium on Criminal Justice Health. 2022.

[8] The COVID Prison Project. Covid Prison Project–Policy Data [Internet]. 2020 [cited 2022 Oct 13]. Available from: https://covidprisonproject.com/policy-data/

[9] Colorado Department of Corrections Responses to Joint Budget Committee Hearing Questions [Internet]. 2020 [cited 2022 Oct 13]. Available from: https://collective.coloradotrust.org/wp-content/uploads/sites/2/2021/05/20201229_cdoc_covid-19_report.pdf

[10] Jamison P. D.C. jail coronavirus lockdown: Inmates confined to their cells 23 hours a day for a year—The Washington Post [Internet]. [cited 2022 Oct 13]. Available from: https://www.washingtonpost.com/dc-md-va/2021/04/19/dc-jail-lockdown-covid/

[11] Grassian S, Friedman N. Effects of sensory deprivation in psychiatric seclusion and solitary confinement. International journal of law and psychiatry. 1986 Jan 1;8(1):49–65. doi: 10.1016/0160-2527(86)90083-x 3940165

[12] Hagan BO, Wang EA, Aminawung JA, Albizu-Garcia CE, Zaller N, Nyamu S, et al, Transitions Clinic Network. History of solitary confinement is associated with post-traumatic stress disorder symptoms among individuals recently released from prison. Journal of Urban Health. 2018 Apr;95:141–8. 28281161 10.1007/s11524-017-0138-1 PMC5906377

[13] Reiter K, Ventura J, Lovell D, Augustine D, Barragan M, Blair T, et al. Psychological distress in solitary confinement: Symptoms, severity, and prevalence in the United States, 2017–2018. American journal of public health. 2020 Jan;110(S1):S56–62. doi: 10.2105/AJPH.2019.305375 31967876 PMC6987940

[14] Smith PS. The effects of solitary confinement on prison inmates: A brief history and review of the literature. Crime and justice. 2006 Jan;34(1):441–528.

[15] Kaba F, Lewis A, Glowa-Kollisch S, Hadler J, Lee D, Alper H, et al. Solitary confinement and risk of self-harm among jail inmates. American journal of public health. 2014 Mar;104(3):442–7. doi: 10.2105/AJPH.2013.301742 24521238 PMC3953781

[16] Williams BA, Li A, Ahalt C, Coxson P, Kahn JG, Bibbins-Domingo K. The cardiovascular health burdens of solitary confinement. Journal of General Internal Medicine. 2019 Oct;34:1977–80. doi: 10.1007/s11606-019-05103-6 31228050 PMC6816726

[17] Brinkley-Rubinstein L, Sivaraman J, Rosen DL, Cloud DH, Junker G, Proescholdbell S, et al. Association of restrictive housing during incarceration with mortality after release. JAMA network open. 2019 Oct 2;2(10):e1912516–. doi: 10.1001/jamanetworkopen.2019.12516 31584680 PMC6784785

[18] Haney C. The psychological effects of solitary confinement: A systematic critique. Crime and Justice. 2018 Mar 1;47(1):365–416.

[19] 70/175. United Nations Standard Minimum Rules for the Treatment of Prisoners (the Nelson Mandela Rules) [Internet]. 2015 [cited 2022 Dec 25]. p. 1–33 Resolution adopted by the General Assembly of the United Nations. Available from: https://cdn.penalreform.org/wp-content/uploads/1957/06/ENG.pdf

[20] Maycock M. ‘Covid-19 has caused a dramatic change to prison life’. Analysing the impacts of the Covid-19 pandemic on the pains of imprisonment in the Scottish Prison Estate. The British Journal of Criminology. 2022 Jan 1;62(1):218–33.

[21] Gray R, Rooney B, Connolly C. Experiences of COVID-19 isolation in Northern Ireland prisons: a qualitative study. International journal of prisoner health. 2021 Oct 18;17(3):304–19.

[22] Sorge A, Bassanini F, Zucca J, Saita E. “Fear can hold you, hope can set you free”. Analysis of Italian prisoner narrative experience of the COVID-19 pandemic. International Journal of Prisoner Health. 2021 Oct 18;17(3):406–23. doi: 10.1108/IJPH-07-2020-0051 34383394 PMC8753627

[23] Liu YE, LeBoa C, Rodriguez M, Sherif B, Trinidad C, Rosario MD, et al. COVID-19 policies in practice and their direct and indirect impacts in Northern California jails. medRxiv. 2022 Jan 12:2022–01.

[24] Pyrooz D.C., Labrecque R.M., Tostlebe J.J. and Useem B., 2020. Views on COVID-19 from inside prison: Perspectives of high-security prisoners. Justice Evaluation Journal, 3(2), pp.294–306.

[25] Johnson L, Gutridge K, Parkes J, Roy A, Plugge E. Scoping review of mental health in prisons through the COVID-19 pandemic. BMJ open. 2021 May 1;11(5):e046547. doi: 10.1136/bmjopen-2020-046547 33986064 PMC8727680

[26] Puglisi LB, Brinkley-Rubinstein L, Wang EA. COVID-19 in carceral systems: a review. Annual Review of Criminology. 2023 Jan 27;6(1):399–422.

[27] Ahalt C, Sudore R, Bolano M, Metzger L, Williams B. “Teach-to-goal” to better assess informed consent comprehension among incarcerated clinical research participants. AMA journal of ethics. 2017 Sep 9;19(9):862. doi: 10.1001/journalofethics.2017.19.9.peer3-1709 28905727 PMC5762181

[28] Smoyer AB, Blankenship KM, Belt B. Compensation for incarcerated research participants: diverse state policies suggest a new research agenda. American Journal of Public Health. 2009 Oct;99(10):1746–52. doi: 10.2105/AJPH.2008.148726 19696389 PMC2741529

[29] Divya R, Christopher PP, Filene EJ, Reifeis SA, White BL. Financial Payments for Participating in Research while Incarcerated: Attitudes of Prisoners. IRB: Ethics & Human Research. 2018 Nov;40(6):1–6.

[30] Yale University Human Research Protection Program. Policy and Standard Operating Procedure Manual. Version 1.1. July 24, 2023. Available at: https://your.yale.edu/sites/default/files/files/ResearchSupport/HRPP/HRPP%20Policy%20and%20Standard%20Operating%20Procedure%20Manual_20230724.pdf

[31] Puglisi LB, Rosenberg A, Credle M, Negron T, Martin RA, Maner M, et al. Paths to improving pandemic preparedness in jails and prisons: perspectives of incarcerated people and correctional staff. American Journal of Public Health. 2022 Nov;112(S9):S869–73. doi: 10.2105/AJPH.2022.306956 36446054 PMC9707706

[32] Braun V, Clarke V. What can “thematic analysis” offer health and wellbeing researchers?. International journal of qualitative studies on health and well-being. 2014 Jan 1;9(1):26152. 25326092 10.3402/qhw.v9.26152 PMC4201665

[33] Deterding N. M., & Waters M. C. (2021). Flexible coding of in-depth interviews: A twenty-first-century approach. Sociological methods & research, 50(2), 708–739.

[34] Bottoms AE. Interpersonal violence and social order in prisons. Crime and justice. 1999 Jan 1;26:205–81.

[35] Noonan M, Rohloff H, Ginder S, International R. Mortality in Local Jails and State Prisons, 2000–2013—Statistical Tables. U.S. Department of Justice Office of Justice Programs. 2015 Aug. https://www.ojp.gov/ncjrs/virtual-library/abstracts/mortality-local-jails-and-state-prisons-2000-2013-statistical

[36] Cramer RJ, Wechsler HJ, Miller SL, Yenne E. Suicide prevention in correctional settings: Current standards and recommendations for research, prevention, and training. Journal of Correctional Health Care. 2017 Jul 1;23(3):313–28. doi: 10.1177/1078345817716162 28656789

[37] Cloud DH, Ahalt C, Augustine D, Sears D, Williams B. Medical isolation and solitary confinement: balancing health and humanity in US jails and prisons during COVID-19. Journal of General Internal Medicine. 2020 Sep;35(9):2738–42. doi: 10.1007/s11606-020-05968-y 32632787 PMC7338113

[38] Esposito M, Salerno M, Di Nunno N, Ministeri F, Liberto A, Sessa F. The risk of COVID-19 infection in prisons and prevention strategies: a systematic review and a new strategic protocol of prevention. Healthcare 2022 Jan 29; 10 (2): 270, MDPI. doi: 10.3390/healthcare10020270 35206884 PMC8872582

[39] Time-In-Cell: A 2021 Snapshot of Restrictive Housing based on a Nationwide Survey of U.S. Prison Systems. 2022 Aug. Correctional Leaders Association and Arthur Liman Center for Public Interest Law at Yale Law School. https://law.yale.edu/sites/default/files/area/center/liman/document/time_in_cell_2021.pdf

[40] Aranda M. National monitoring bodies of prison conditions and the European standards. Rome. Antigone Edizioni. 2015 Jan. Available at: http://www.prisonobservatory.org/upload/National%20monitoring%20and%20EU%20standards.pdf. 2015.

