# Supplementary material for: “It’s just us sitting there for 23 hours like we done something wrong”: Isolation, incarceration, and the COVID-19 pandemic
Source: PLoS One. 2024 Feb 14;19(2):e0297518. doi: 10.1371/journal.pone.0297518 (PMC10866499; doi:10.1371/journal.pone.0297518)
Supplement: S1 File — (DOCX) [file pone.0297518.s001.docx]

**SI1 Interview Guides**

**Interview guide for incarcerated participants**

*General:* To get started, why don’t you tell me a little about yourself.

- Where are you from originally?
- What is your day to day routine?
- How long have you been incarcerated? How much longer do you expect to be incarcerated? How long have you been at this facility?

*General COVID Experience:* What has it been like to be incarcerated during the COVID pandemic? What adjustments have you had to make?

- What are your thoughts about COVID? What do you believe about it? Does it even exist? Tell me about that.
- How are things different now than before? Are there any changes? (probe for changes to visitation, amount of isolation/lockdown) Do you have any stress about COVID? =If so, how do you cope with your stress about COVID?
- Tell me about safety measures like mask wearing and keeping a six-foot distance (probe for incarcerated people and staff)
- What happens when someone tests positive? How do you feel about the care being provided to COVID positive patients here?
- How have the Correctional Officers reacted to COVID? Do you get a sense of how they feel about testing/vaccine?
- Has anything changed about the length of your incarceration or sentencing? Have you heard of others getting released early? Can you tell me about that process? What about compassionate release?

*Medical care experience at facility:* What is your relationship like with the medical staff at this facility?

Probe for examples:

- Have you needed medical care during this incarceration?
- How have medical staff responded to your need for care?
- Do you have the opportunity to discuss questions about COVID with medical staff, for example about testing? What about vaccination?

*Experience with COVID 19 testing:* Have you been tested for COVID in prison/jail? What was that like? Can you describe the procedure? What are your thoughts about testing?

- How often are incarcerated people tested here?
- Do people have a choice about getting tested or not? Should it be mandated or voluntary? Can you tell me about that?
- Are people tested/can people request a test when they are symptomatic? Have you ever requested a test? What happened?
- Do they let you know the result of the test? If so, what is the waiting period for the result? How are you told?
- What do you think about the COVID testing happening? Are there good things about being tested and the way the testing is done? What about negative things? What would you change about COVID testing if you could? What would prevent someone from getting tested? Probe for different areas, including frequency of, choice/no choice, wait time to results, how told, consequences of.
- Have you tested positive for COVID during your incarceration? Do you know anyone that has tested positive? What was that experience like for you/him/her?
- Have you or anyone you’ve known been hospitalized for COVID? Has anyone you’ve known lost their life from COVID? What was the notification process like (for both hospitalization and death)?
- How important do you think testing is for controlling COVID in prison/jail?
- Who do you think should have access to your test results? Who DOES have access to your results? (probe for sharing within the facility (people you live with), community (family, probation/parole/halfway house), government/other agencies.
- What would be the best way to go about testing in correctional facilities?
- What advice do you have for those working on testing strategies in prison/jail?

*Treatment:* What happens when you test positive for COVID here?

- If you test positive, what treatment is given? (If personal experience of testing positive can revisit: How was treatment? Where were you treated? Also probe for what happens to your housing, release plans, job. What happens to your entire housing unit? What happens when there are a lot of COVID cases within a housing unit?
- Would you change anything about how COVID positive patients are treated here?

Now we want to talk now about the COVID vaccine. But before starting, how do you feel about vaccines in general? Can you tell me about that?

*Vaccine:* How do you feel about the COVID vaccine?

- What goes into your decision about getting vaccinated? Can you share your thoughts about the advantages to getting vaccinated? What about disadvantages? If a vaccine is offered to you in prison/jail, will you choose to get vaccinated?
- Would your willingness to get vaccinated be different if you were not incarcerated? Please explain.
- Do you think vaccines in correctional settings should be required or voluntary? Please explain.
- What would prevent someone from getting a vaccine for COVID in prison and jail?
- What would encourage someone to get vaccinated in prison or jail? (could probe for incentives, correctional officer vaccination alongside, more information)
- Where are you getting information about the vaccine? Do you feel you have received enough information about the vaccine?
- Have you been vaccinated for any other illness while incarcerated? What was that like?

*General:* How has your experience with COVID compared with other experience of illness in prison/jail?

- Probe for testing, vaccination and treatment.

Research:

- What are your thoughts about participating in research while incarcerated?
- What about clinical trials? (A clinical trial is a research study to test a drug or vaccine to see how well it works. Sometimes the participant gets the actual drug or vaccine, and sometimes they get a placebo or control.)
- Would you participate in a COVID vaccine trial while incarcerated? Would this be the same or different than your decision to participate in research while not incarcerated? What would go into your decision to participate in clinical trials while incarcerated?

**Interview guide for correctional officers**

**Introduction:** How long have you been working at XX facility? What has it been like working at XX facility during COVID?

- What are your concerns/worries? How is working here different now than before?
- To what extend do you feel at risk of getting COVID at work? What puts you at risk?
- What has it been like going into the facility and back to your family and community each day? How do you deal with that? What does your family think?
- Have social distancing and mask wearing rules been implemented and if so, what are they like? To what extent are these measured followed?

**Testing:** Let’s talk about COVID testing at this facility. Can you walk me through the process of getting tested for correctional officers? What about for incarcerated people? Probe for both populations: frequency, COs and incarcerated people, choice, results, waiting period, treatment, prevention. Probe for both populations:

- Have you been tested? What was that like?
- How willing are people to get tested? Why?
- Do you think tests should be mandated or voluntary? Why?
- What about privacy and confidentiality when it comes to testing?
- If you could change anything about testing at this facility, what would you change?

**Vaccination:** Let’s talk about vaccination for COVID at this facility. We understand that vaccines are/will be available for those that work here and live here. Will you/have you gotten a vaccine? Why/why not? Probes for both populations:

- Why do some people get vaccinated and some don’t? What do you think would help more people get vaccinated? Probe if needed for incentives, choice, education, treatment
- How willing are people to get vaccinated?
- Do you think the vaccine should be mandated or voluntary? Why? Any worries about vaccine roll out in this facility?
- What about privacy and confidentiality when it comes to vaccination?
- If you could change anything about vaccination at this facility, what would you change?

**Changes in work:** How has COVID changed the work of being a CO? Are there different situations you have to handle here now? Are there differences in how disciplinary situations are handled?

**Union:** Are you a part of a union? How does that impact COVID related concerns/protections? Do you feel the union is supporting you?

**Wrap-up:** What has helped make you feel safe during COVID? What thinks make it feel more dangerous?

**Interview Guide for correctional leaders**

*General:* What is your position at XX facility? What are your responsibilities?

*COVID Role:* What has your role been in the response to the COVID pandemic in this facility/state’s facilities?

- What goes into your decisions about protocols for COVID?
- What has been the most challenging part of making or implementing COVID related changes?
- Are there things that are out of your control that you wish could be changed about COVID in this facility? Are there places of conflict in COVID protocol decisions that you can talk about, for example, where resources don’t match protocols? Probe for financial, political climate
- How are you working with your medical partner to implement COVID protocols?

*Testing:* Can you walk me through the COVID testing procedure here for correctional staff? What about for incarcerated people? How is it working?

- How has that changed over time? Why? What are the current challenges with the strategy for testing in facilities?
- How important do you think testing is for controlling COVID in prison?
- What has COVID testing acceptance been like? What would increase acceptance?
- How is privacy/confidentiality protected when it comes to COVID testing? Do you have any concerns about privacy/confidentiality when it comes to testing? What about data sharing?
- Do you think testing should be mandated or voluntary? Why?
- What would you change about the current testing policy, if you could change it? Why?
- What do you think incarcerated people think about testing in prison? What about correctional staff? If concerns, why, and what would ease their concerns?

*Vaccine:* What is the status of COVID vaccinations at [facility]? Do you have ideas about how to successfully rollout the vaccine within facilities (for both correctional staff and incarcerated people)?

- Do you have any suggestions for what they could have done differently, instead of the mandate, for increasing the vaccination rate among correctional officers?
- Do you have any worries about vaccine roll out in this facility?
- How willing do you think people will be to get it? Incarcerated people? Correctional staff? Can you explain?
- What do you think would prevent someone from getting a vaccine for COVID -19 in prison and jail?
- What would encourage someone to get vaccinated in prison or jail? (Could probe for mandated, incentives, correctional officer vaccination alongside, more information).
- Successes and failures of vaccination efforts to date.

*Treatment:* What happens when someone tests positive here (treatment, prevention, transportation, housing unit, hospitalization)? Would you change anything about how COVID positive patients are treated here?

*General:* Can you think of other health initiatives or challenges in correctional settings that could inform decisions about COVID testing or vaccine implementation in facilities here? What other experiences will inform your decisions about testing or vaccine implementation in the future?
